# The Dual Role of Nanomaterials in Ovarian Cancer and Female Fertility as Anti- and Prooxidants

**DOI:** 10.3390/antiox14091066

**Published:** 2025-08-29

**Authors:** Massimo Aloisi, Gianna Rossi, Sandra Cecconi

**Affiliations:** Department of Life, Health and Environmental Sciences, University of L’Aquila, 67100 L’Aquila, Italy; massimo.aloisi@univaq.it (M.A.); gianna.rossi@univaq.it (G.R.)

**Keywords:** ovarian cancer, female fertility, nanomaterials, oxidative stress

## Abstract

Nanomaterials (NMs) are becoming increasingly important in biomedical applications, especially in reproductive biology and oncology. In this review, we examined the “double face” of NMs as prooxidants and antioxidants in relation to ovarian cancer and female fertility. NMs have been shown to reduce oxidative stress pathways in tumors, enhancing the effectiveness of chemotherapy and serving as carriers for drugs and compounds. They are also considered for their protective effects on female fertility by improving oocyte quality, maturation, and survival under various healthy and adverse conditions. However, certain NMs can induce oxidative stress, mitochondrial dysfunction, and ovarian tissue apoptosis when present in high concentrations or after prolonged exposure. These “double face” effects highlight the complex nature of NMs’ concentration, shape, and biocompatibility. Although NMs show promise in cancer treatment and fertility preservation, a comprehensive assessment of their prooxidant potential is necessary for successful clinical application.

## 1. Introduction

### 1.1. Nanomaterials Generalities

Nanomaterials are defined as “*natural, incidental, or manufactured materials containing particles, in an unbound state or as an aggregate or as an agglomerate and where for 50% or more of the particles in the number size distribution one or more external dimensions is in the size range 1–100 nm*” [1].

NMs have unique chemical, physical, and biological properties and can be classified based on shape, composition, size, and origin [2]. Starting with their origin, they are grouped into naturally occurring and artificial NMs [3]. Naturally occurring NMs come from natural events like volcanic eruptions, fires, dust storms, and from general environmental sources [4]. Even when only produced sporadically in specific areas, they pose a risk to humans; for example, volcanic ash has been shown to carry toxic metals, increasing their toxicity [5]. Additionally, naturally occurring NMs are relevant to occupational risks, such as silica NMs causing lung toxicity and DNA damage in miners [6,7]. Artificial NMs are created through industrial or laboratory processes and can be either intentionally or unintentionally produced. Intentional NMs are added to products for specific purposes, such as in cosmetics like scrubs (microplastics), sunscreens (titanium dioxide, TiO_2_), shampoos, and others, as well as in electronics and packaging [8]. Incidental NMs are not deliberately made but are released as a byproduct of human activities; examples include the NMs in vehicle and industrial emissions [9,10]. Because of their presence in the environment, it is essential to evaluate their biological effects.

Size is the second most important feature because it can influence NM properties. This is evident from the use of silver (Ag) NMs to color church windows, a technique used since the Middle Ages, as they reflect light at different wavelengths depending on their size [11]. Today, several NMs are employed for imaging purposes due to their dimensions and ability to emit fluorescence, such as quantum dots [12]. Size is also crucial for understanding the kinetics of NMs in the environment and in biological samples. Smaller particles can float and typically do not settle after Brownian motion; in contrast, larger particles tend to settle and accumulate on ocean floors and in soils. Therefore, depending on animal habits, they follow different exposure routes that need to be considered [13,14]. In contrast to microparticles, NMs have a higher surface/volume ratio, and small differences can impact their properties [15]. Smaller NMs can go through biological barriers such as the cell membrane, blood–brain barrier, or placenta via passive and facilitated diffusion or endocytic transport [16]. This implies that NMs can be biodistributed differently to micromaterials and reach deeper tissues [16]. Furthermore, size influences the way NMs are internalized. Smaller particles are mainly absorbed via caveolae- or clathrin-mediated endocytosis, while larger particles are absorbed via phagocytosis or macropinocytosis. This has direct consequences on the speed with which they enter the cell, on their cellular targets, and on the cellular response [17]. From a pharmacological point of view, smaller NMs can exploit this property to reach tumors that are screened by biological barriers and that are more difficult to be targeted [18]. This same property may induce more toxic effects in nontumoral cells, but this issue could be avoided by coating NMs with specific antibodies, as discussed later in this review [19,20]. Size also influences the formation of the protein corona (discussed in the next paragraph) and the possibility of using NMs as vectors [21]. In this case, smaller NMs could have, because of their higher curvature, a reduced capability for compound binding compared to bigger particles. Therefore, every case and need should be evaluated carefully [21]. In addition, size seems to be correlated with the genotoxicity of some NMs and the reactive oxygen species (ROS) generated. These results indicate that higher cellular absorption can lead to higher toxic effects. Therefore, the size-dependent effects could be used to increase damage in cancerous cells but could also lead to higher damage to healthy cells [22,23,24].

Particles can interact to form larger structures that alter their kinetic properties and can also react with molecules from their surroundings or biological fluids. When NMs bind metals or organic molecules, they can transport these substances, acting as carriers. This process, known as the “Trojan horse”, increases the level of pollutants that living organisms are exposed to [25].

In biological systems, smaller NMs can cross biological barriers and reach deeper tissues, increasing toxicological risks [26]. Recently, this property has been used to deliver drugs and compounds to tissues and to boost antibiotic effectiveness [27,28]. In mammalian tissues, NMs can bind to proteins and molecules, forming an external layer called the “protein corona”, which is able to increase size and to alter surface chemical properties of NMs [29].

Shape directly influences NMs’ toxicity and the applications they can be used for [30]. While spherical NMs are generally considered less harmful than sharp ones, this is not always true. For instance, gold (Au) nanospheres were more toxic to human renal cells than Au nanostars, and flower-shaped AuNMs, with their rough surfaces, showed increased toxicity compared to spherical NMs [31,32]. Additionally, both silicon (SI) and zinc oxide (ZnO) NMs caused more oxidative stress and DNA damage when they were sharp compared to when they were spherical [33,34]. Despite this, spheres are usually taken into cells more easily than rods, suggesting that shape is more important than concentration in determining NM toxicity [35]. Therefore, NMs used in biomedical applications, such as cancer treatments, need to be carefully chosen to enhance their effectiveness.

The last feature to be considered is the chemical composition of NMs, which allows for the identification of both organic and inorganic NMs. Organic NMs include liposomes, dendrimers, carbon-based particles, and various polymers; inorganic NMs consist of any chemical element, such as silver (Ag), Au, Zn, and others [36,37]. The chemical composition of NMs affects their reactivity with biological systems. For example, gold is considered non-reactive, making it one of the most promising NMs in clinical practice [38]. It can also be modified by functionalizing NMs with molecules, such as compounds for transport, similar to an intentional Trojan horse, as previously described [39,40]. Thanks to their lipidic structures, micelles and liposomes are the standard NMs used to carry compounds to increase their absorbance [40]. Moreover, adding peptides to these NMs was proven to further increase their absorption and specificity to HER2-Positive Breast Cancer [41]. Additionally, negatively charged NMs tend to be absorbed less due to electrostatic repulsion with cellular membranes [42]. These characteristics, among others, are considered when NMs are used. In Table 1, we summarize the main NMs and their most common biomedical applications. The table is not meant to report all the existing NMs, but rather the ones discussed in this review and, even if not referred to, the most relevant ones nowadays.

### 1.2. Oxidative Stress

NMs such as fullerenes, platinum, Au, and cerium oxide NPs (CNPs or nanoceria) have antioxidant properties that make them suitable for conditions characterized by oxidative stress (OS).

**Figure 1:**
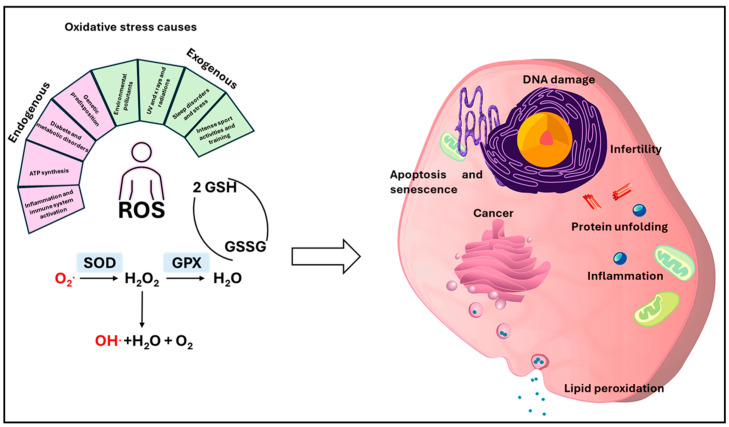
Causes and consequences of oxidative stress. Oxidative stress can be induced by endogenous and exogenous causes. Both lead to the generation of molecules containing oxygens with an unpaired electron. This characteristic makes them able to oxidize other biological molecules, leading to disruption of their function. Biological organisms have developed physiological mechanisms to control this phenomenon; these mechanisms involve enzymes such as SOD and GPx and molecules like vitamins E and C. The balance between these processes is fundamental to avoiding cellular toxicity. Glutathione peroxidase (GPX), Superoxide dismutase (SOD), Glutathione (GSH), Glutathione disulfide (GSSG) (picture designed with Pixabay).

OS is defined as “an imbalance between oxidants and antioxidants in favor of the oxidants, leading to a disruption of redox signaling and control and/or molecular damage” [99] (Figure 1). Therefore, the cause of the adverse effects following OS is an unbalanced cellular environment involving the molecular pathways needed to detoxify reactive molecules or repair their damage and the generation of new reactive oxygen species (ROS). Considering that free radicals react with proteins, DNA, and lipids, this alteration damages cells and tissues [100]. Normally, ROS are naturally produced during physiological metabolic processes, particularly in mitochondria. To maintain a healthy balance, endogenous antioxidant molecules stop the oxidative cascade, providing new electrons to free radicals. However, when free radicals overwhelm antioxidant defenses [101], OS contributes to the development and progression of several chronic and degenerative diseases, such as diabetes, cancer, cardiovascular disease, neurodegenerative diseases like Parkinson’s and Alzheimer’s, and inflammatory conditions. This proinflammatory state accelerates aging in most tissues [102,103]. Environmental factors like pollution, cigarette smoke, and UV light can exacerbate OS. Considering all of these outcomes, OS is generally labeled as harmful, but it can also be used therapeutically, such as in cancer treatment, thanks to its effects on cell signaling and the immune response [104]. Scavenging molecules include enzymes such as glutathione peroxidase (GPx), peroxiredoxins, sulfiredoxin, catalase (CAT), and superoxide dismutase (SOD) and non-enzymatic antioxidants like glutathione and vitamins A, C, and E [105]. Since some NMs have antioxidant abilities as well as their capacity to transport compounds, their potential use for treating various pathologies generates new therapeutical opportunities.

### 1.3. Female Reproductive Health and Oxidative Stress

The female reproductive system is particularly sensitive to OS because of its physiology and metabolic activity [106]. In normal conditions, OS is considered a promoter of ovulation [107]. An increase in reactive oxygen species is associated with meiosis I, while antioxidants promote meiosis II [107]. Also, ovulation is stimulated by a luteinizing hormone surge that generates inflammatory precursors, causing OS. Depleting these precursors impairs ovulation [108]. These results highlight how a well-modulated redox balance is essential for fertility: controlled ROS production promotes oocyte maturation and egg release, while an excess or deficiency can compromise reproductive function. With aging, egg quality declines, partly due to the accumulation of oxidative damage, especially in both nuclear and mitochondrial DNA. After menopause, the reduction in estrogen and the increase in iron stores further contribute to OS; there are also cardiovascular implications [109]. The environment has an important role, too. It can increase the amount of OS, impacting female reproductive health. Smoking generates ROS and depletes antioxidants, compromising fertility and increasing the risk of miscarriage [110]. Alcohol produces acetaldehyde and ROS, activating inflammatory pathways and damaging the placenta and fetus [111]. Drugs such as cannabinoids and cocaine generate ROS and cause damage to DNA and mitochondria, with negative effects on pregnancy [112]. Organochlorine pesticides such as DDT, PCBs, and organophosphate compounds accumulate in tissues and follicular fluids, altering reproductive function and increasing OS. Chronic exposure to these agents can also compromise fetal development [113]. Considering the delicate balance between OS and antioxidant pathways and physiological conditions, the role of external agents is gaining more and more relevance. Impairments of this balance contribute, as described, to altering reproductive health.

The main goal of this review is to describe the role that NMs may play in treating OS imbalances in health and disease. In particular, we will focus on two of the most common conditions: ovarian cancer (OC) and infertility. In both cases, we will describe only the pro- and antioxidant NMs and their ability to ameliorate OS and the related disease or their ability to induce OS, which can be exploited against OC or considered as a negative outcome in gamete preservation

### 1.4. Methodologies

In the next two sections, we will discuss some recent results obtained on OC and female infertility. To do so, we reviewed the scientific literature without a specific time frame; we considered publications from any year, trying to balance historical relevance and the most recent publications. No exclusion methods were used. We apologize if we have missed some relevant work. We found references from the most important databases: Pubmed and Scopus. We used keywords such as “antioxidant nanomaterials”, “ovarian cancer”, “oxidative stress and female reproductive health”, “nanomaterials and female health” and combinations of the presented keywords.

## 2. NMs and Ovarian Cancer (OC)

OC is characterized by delayed diagnosis because of its symptoms, which are difficult to distinguish from other conditions. Over 70% of OCs are diagnosed at stage III or IV, meaning survival rates are low [114]. To add complexity, many OCs begin in the fallopian tubes rather than the ovaries [114]. Around 240,000 new cases of OC are reported each year, making it the seventh most common cancer in women worldwide. There are 14,000 fatalities and 22,000 new cases annually in the US alone [115]. Among the various types of OC, the most common is epithelial serous carcinoma, which is divided into high-grade (HGSC) and low-grade (LGSC) histologic subtypes [116]. OC occurs occasionally, but several risk factors increase susceptibility, such as genetic predisposition, ovulation cycles, Lynch syndrome, endometriosis, dietary factors, ethnicity, and mutations in the BRCA1, BRCA2, and MMR genes [117]. The two main treatment options are surgery and chemotherapy, which usually consists of carboplatin and paclitaxel (PTX). Carboplatin is an anti-cancer drug belonging to the class of platinum compounds. It is a DNA-binding molecule that is able to prevent cell replication and proliferation. Its mechanism of action is based on its ability to form covalent bonds with DNA bases, causing a distortion of the double helix and blocking DNA transcription and replication. In ovarian cancer, which is characterized by fast growth, carboplatin acts on fast-dividing cells. Once administered, carboplatin enters the cells and, thanks to its chemical structure, is activated, forming a reactive compound. This compound binds to DNA and creates intra- and inter-DNA cross-links that prevent the cell from correctly reading the genetic code. The result is a blockage of cell division and the activation of programmed death mechanisms, such as apoptosis [118]. Paclitaxel is an anti-cancer drug belonging to the taxane class that was first isolated from the Pacific yew tree (Taxus brevifolia). Its mechanism is related to interfering with the division of cancer cells by blocking the function of microtubules that are essential for mitosis. Paclitaxel binds to tubulin and stabilizes microtubules, preventing their disassembly. This blockage stops the cell in the G2/M phase of the cell cycle, causing mitosis to stop and, consequently, programmed cell death (apoptosis) to start [119].

Combining chemotherapy with other treatments, like PARP inhibitors, can have a synergic effect, increasing treatment efficacy. Also, it has been proven that intraperitoneal chemotherapy improves survival rates in advanced cases by increasing concentrations at the tumor site and lowering systemic toxicity [120]. Therapies aimed at new targets to reduce side effects are crucial to overcome chemoresistance and improve personalized medicine [121]. NMs are ideal for this purpose thanks to the fact that they can be used both as compound vectors and bioactive agents. To reduce side effects, antibodies can be used to lead the NMs to the tumor site [121]. Guiding antibodies and anti-cancer compounds can both be used on the same nanoparticle, highlighting the incredible number of possibilities that these technologies allow. For instance, Jain et al. [122] explored the efficacy of paclitaxel encapsulated in a poly(propylene imine) (PPI) dendrimer conjugated with an antibody specific to mesothelin, which is highly expressed in ovarian cancer. The system allows the compound to be transported only to malignant cells, avoiding side effects. In both the OVCAR-3 cell line (MTT assay, 0.01 μM to 10 μM) and mice (10 mg/kg), researchers observed a higher benefit in terms of tumor reduction with fewer side effects. Furthermore, the introduction of green synthesis, which starts from biological compounds to produce NPs, has reduced the toxicity of NM production [123].

ZnO, AgNPs, and selenium (Se) can alter the expression of genes related to OS in A2780 ovarian cancer cells, which is linked to antioxidant and anticancer properties [124]. In vitro studies showed that upregulating the SOD2 gene and downregulating the NOX4 gene, which is involved in the production of ROS, affected tumor cell proliferation. Consequently, ZnO, Se, and AgNPs are promising therapeutic agents and NOX4 and SOD2 could be considered as biomarkers. Innovative PTX-loaded zein nanoparticles (NanoPTX) proved to efficiently target hHGS-OC cells and to increase cell death in comparison to PTX alone, but PTX and NanoPTX did not have a significant effect on the viability of COV 362 cells [125]. Chen et al. [126] studied the green synthesis of gold NPs (AuNPs) and assessed their cytotoxic, antioxidant, and anti-OC properties using an aqueous extract of Curcumae Kwangsiensis Folium leaves. Nanoparticles (8 to 25 nm) showed cytotoxic effects on the PA-1, SW-626, and SK-OV-3 cell lines, decreasing cell viability in a dose-dependent manner. Moreover, they had no cytotoxic effects on normal human endothelial cells (HUVEC), indicating that they could have a specific impact only on tumor cells. This result is interesting considering that no active targeting was present. Lastly, with an IC50 value of 153 µg/mL, they acted as better antioxidants than butylated hydroxytoluene (BHT), which is often used as an antioxidant control. Sindhu et al. [127] examined the therapeutic potential of flavonoid-based nano-systems for breast cancer treatment. Their results suggest that flavonoids’ antioxidant and anticancer properties may help to regulate tumor-related metabolism and inhibit cell growth. Epidemiological studies have linked high dietary flavonoid intake to a decreased risk of breast cancer, especially in postmenopausal women [128]. Other flavonoids, such as quercetin, genistein, apigenin, and epigallocatechin-3-gallate, have shown anti-tumor effects by triggering apoptosis, preventing angiogenesis, and altering cell signaling pathways [129]. Despite this, their therapeutic application is limited by their low bioavailability in the gastrointestinal tract. Nanotechnologies, such as metallic NPs, lipid NPs, and nano-capsules, have been generated to address this issue. Flavonoid-based cancer treatments are more effective thanks to these nano-formulations, which increase drug solubility and bioavailability [130,131,132,133,134]. A less-used type of NM is gaining attention. The potential biomedical uses of CeO_2_NPs, due to their anti-inflammatory, neuroprotective, and anti-cancer properties, have been published [135]. By lowering ROS, they demonstrated antioxidant qualities. Indeed, they can neutralize free radicals such as hydrogen peroxide and superoxide anions thanks to the redox cycle between the Ce^3+^ and Ce^4+^ states, similar to the way that the enzymes CAT and SOD prevent oxidative damage in cells. New techniques have recently been developed to analyze the antioxidant properties of ceria by altering its surface or increasing its catalytic activity [136]. Varukattu et al. [137] investigated the therapeutic potential of hyaluronic-acid-functionalized poly(ethylenimine)-nanoceria (HA-CePEI-NPs) as a nanoreactor for the treatment of triple-negative breast cancer. HA-CePEI-NPs slowed the growth of cancer cells, inducing cell cycle arrest in the G2/M phase. Compared to non-functionalized nanoceria, they increased therapeutic efficacy by exhibiting increased cellular uptake through the CD44 receptor. Adebayo et al. [138] used female Wistar rats as a breast cancer model, exposing them to benzo(a)pyrene (BaP) and N-methyl-N-nitrosourea (NMU) to show the anti-tumor effects of ceria. SOD, CAT, glutathione-S-transferase, and other antioxidant enzyme activities were significantly reduced after exposure to both contaminants. OS, inflammation, and lipid peroxidation were also increased. Rats exposed to NMU and BaP showed decreased apoptosis related to the downregulation of Bax, p53, and caspase-3. In addition, by reducing inflammation and OS, CeO_2_NP treatment restored antioxidant enzyme levels.

**Figure 2:**
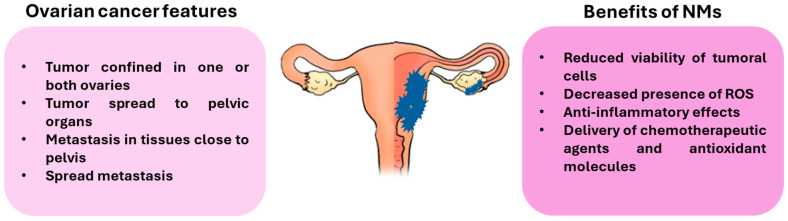
The main features of OC and the benefits of NMs. The use of NMs is gaining attention in treating OC. Thanks to antibodies, NMs can specifically target malignant cells, releasing anti-cancer drugs specifically in those cells. Moreover, NMs can be used as vectors for antioxidant compounds that are useful in reducing tumor progression (picture designed with Pixabay).

All these results confirm that NMs can help breast cancer chemotherapy by increasing drug and compound efficacy, although further studies and clinical trials are needed to translate the results from cells and animals to humans (Figure 2).

## 3. NMs and Female Fertility

**Figure 3:**
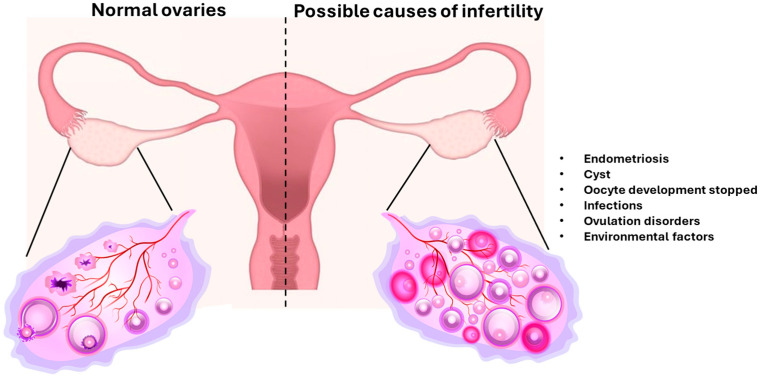
Female infertility causes. The presence of pathological conditions can increase the probability of infertility. Endometriosis, cysts, infections, environmental pollution, and health conditions are just some of many relevant factors (picture designed with Pixabay).

The presence of a small, predetermined number of oocytes in the ovary makes female fertility a delicate balance between hormones, the environment, and overall health (Figure 3). Therefore, infertility can be induced by various pathological conditions (e.g., endometriosis, chromosomal abnormalities, ovulatory disorders, genetic mutations), unhealthy lifestyles (e.g., diet, stress, alcohol use, smoking, obesity), and environmental pollution (e.g., heavy metals, endocrine disruptors, pesticides). Studies have linked exposure to bisphenol A, phthalates, and parabens to a range of health problems because they interfere with the hypothalamic–pituitary–ovarian axis by altering hormone release. These alterations can increase the risk of infertility and fetal abnormalities by disrupting folliculogenesis, ovulation, and pregnancy [139,140].

The ability of NP’s to pass through physiological barriers (blood–testis, placenta) and accumulate in reproductive organs shows the potential risks for both males and females following exposure to NPs. The molecular mechanisms involved are not yet well understood, but they commonly trigger inflammatory responses and genotoxicity. The toxic effects of NPs in female reproduction are based on the ability of NP’s to affect oogenesis and harm fetal development. Despite this, there is a positive aspect linked to the use of NPs, as they can be used as drug carriers or in nanoimaging technologies [141].

### 3.1. Antioxidant Effects

Endometriosis can result from excessive angiogenesis and OS [142]. CeO_2_NPs might help treat this condition and improve female fertility. In a mouse model of endometriosis (using CD-1 strain Swiss Albino mice injected peritoneally with 0.5 mg/kg twice daily for 15 days), nanoceria showed anti-angiogenic and antioxidant effects by significantly lowering vascular endothelial growth factor (VEGF), lipid peroxidation, and ROS levels, and also improving oocyte quality. Nanoceria proved to be better than N-acetyl cysteine, a known antioxidant, at reducing OS and inhibiting angiogenesis. Histological analysis of the treated mice revealed decreased microvascular density and fewer endometrial glands [143]. Another study evaluated the effects of CeO_2_NPs on aged mouse oocytes, focusing on the role of NMs in oocyte maturation and granulosa cell health [144]. In aged Balb/c mice (treated with 45 mg/kg once daily for 3 days), ceria increased the number of mature oocytes and improved granulosa cell survival. However, the same treatment did not cause significant changes in granulosa cells or oocyte maturation in younger mice. Ariu et al. [145] demonstrated the effects of increasing concentrations (0, 44, 88, or 220 µg/mL) of CeO_2_NPs on cumulus-oocyte systems from slaughtered sheep exposed in vitro. The NPs were internalized only in cumulus cells that showed lower mRNA levels related to pro-apoptotic signals and oxidative stress detoxification. Moreover, in vitro-fertilized oocytes exposed to CeO_2_NPs developed into embryos with higher numbers of trophectoderm cells and inner cell mass and greater blastocyst yield.

El-Beltagy et al. [146] found that ZnNPs rescued the toxicity caused by lipopolysaccharide (LPS) in rat placentas and ovaries. Indeed, early exposure of pregnant rats to LPS caused histological alterations. Biochemical analysis showed reduced levels of IGF-1 and an increase in caspase-3, TNF-α, and TGF-β1, proving the presence of OS and inflammatory responses. Treatment with zinc nanoparticles (ZnNPs) from day 14 of gestation successfully reversed these adverse effects by improving tissue integrity and reducing oxidative damage. These results demonstrate how these NPs may help protect reproductive health from bacterial-exposure-related adverse effects.

Also, iron NPs (synthesized with green method synthesis) or bilirubin/melatonin-conjugated glycol chitosan NPs were proven to induce a significant reduction in cytoplasmic ROS levels in oocytes matured in vitro. Both treatments improved oocyte maturation and increased the final number of embryos [147,148]. NMs are useful in cryopreservation technologies. Depending on the process (slow freezing or vitrification), various cryoprotectant agents and other chemicals can be added. Currently, vitrification is the most popular method for preserving germ cells and embryos because of its speed. Unfortunately, the survival rate of vitrified oocytes remains low; however, recent studies show that adding certain types of NPs during the process can improve the protocol. The vitrification of porcine oocytes arrested at prophase I (the germinal vesicle stage) with different concentrations of silicon oxide (SiO_2_), aluminum oxide (Al_2_O_3_), HA, and titanium oxide (TiO_2_) NPs (20 nm diameter) proved to be a promising method. In this experiment, SiO_2_ and HA were the least toxic and most biocompatible. Compared to other treatments and control conditions, these two NMs (used at 0.05%) significantly increased oocyte survival rates and enhanced their ability to complete meiosis up to the metaphase II (MII) stage [149]. To our knowledge, current studies have not highlighted pro- or antioxidant mechanisms related to NMs and cryopreservation. A novel NP made from polylactic-co-glycolic acid and resveratrol was able to reduce ROS levels and increase glutathione levels and the percentage of MII oocytes when added to the culture medium. The antioxidant effect persisted during vitrification, indicating a positive role in improving the overall quality of cryopreserved oocytes [150].

### 3.2. Prooxidant Effects

As previously mentioned, NMs can also have toxic effects on female reproductive tissues. In vitro studies have shown that many NPs can cross theca cell layers and are internalized by granulosa cells, disrupting signaling between the somatic and germinal components [151]. Therefore, it is important to select the right NM for each specific purpose and determine the correct dose in every case. For example, CeO_2_NPs may be used in treating endometriosis, although at high concentrations they exert toxic effects that can alter oocyte development and ovulation [152]. The repro-toxic effects of TiO_2_NPs have been extensively reviewed by us and others [141,153]. Gao et al. [154] proved that intragastric administration of TiO_2_NPs (10 mg/kg) for 90 days in CD-1 (ICR) female mice induced OS, diminishing pregnancy and mating rates, significantly increased E2 and FSH levels, and reduced P4, LH, and T hormones. Histopathological analysis of the ovaries showed the presence of ovarian atrophy and failure. In female NMRI mice, exposure to TiO_2_NPs (100 mg/kg/day, 5 weeks) led to follicle degeneration and cysts, reduced pregnancy rates and embryo development, and significantly increased levels of malondialdehyde, indicating the possible presence of OS [155,156]. Liu et al. [157] studied how SiNPs affected female mice’s ovaries and fertility by giving C57BL/6 mice SiNPs at 0, 3, or 10 mg/kg body weight daily for eight weeks. The results showed that SiNPs were absorbed into ovarian tissue, leading to decreased fertility, hormone imbalance, and follicle depletion. SiNP exposure reduced SOD activity and increased levels of ROS and malondialdehyde. Further analysis revealed that SiNPs caused mitochondrial dysfunction by inducing autophagy through the PINK1/Parkin and PI3K/AKT/mTOR pathways. In addition, the ATM/p53 signaling pathway triggered apoptosis and induced ovarian damage. The addition of N-acetyl cysteine (NAC) decreased OS and restored hormone balance and folliculogenesis, suggesting a protective effect. Zhang et al. [158] reported significant reproductive toxicity after investigating the impact of copper oxide nanoparticles (CuONPs) on mouse oocytes. Exposure to CuONPs affected oocyte maturation by disrupting chromosome stability, kinetochore–microtubule, and meiotic spindle formation. In addition, NM exposure changed the acetylation of α-tubulin, which altered the formation and stability of microtubules. The study also proved that CuONPs altered the cellular location of two proteins that influence fertilization, ovastacin and Juno. OS and mitochondrial dysfunction increased ROS levels, which caused DNA damage and apoptosis.

## 4. Conclusions and Future Prospects

Several studies have shown that NMs can negatively impact female fertility and tissues. However, few of them have focused on OS, mainly because it is not always the primary outcome or priority in these studies. Identifying all the molecular pathways altered after NM exposure is important, especially considering the potential protective effects of NMs and their relevant biomedical applications, as discussed earlier. Moreover, the prooxidant effects of some NMs might be useful in cancer therapy, as using toxic molecules or pathways to target cancer cells is not a new concept. Clarifying the specific pathways that each NM can alter is fundamental to understanding the balance between prooxidant and antioxidant mechanisms. When NMs are absorbed from the environment, it is likely that their effects will lead to increased OS. This information not only helps in understanding the adverse outcomes that living beings are exposed to every day but is also important for possible biomedical applications. Personalized medicine may be able to exploit toxic effects to specifically affect cancerous cells. Therefore, the role of NMs in the general oxidative balance of cells is crucial to deconvolute the possible new applications of standard or new NMs. More and more NMs are generated every day. This is thanks to green synthesis, which is unifying industrial and biomedical needs with ecological ones [159]. It is important to continue testing all new NMs for both toxic and positive properties. New vaccine technologies, lipids for cosmetics, and many more applications represent the new frontiers of NMs research. In addition, NMs represent a new frontier in OC diagnosis and treatment [160,161,162,163,164]. For instance, a nanocombo of small drugs can be used to deliver combinations of more anticancer therapies [155]. Lately, NMs can be generated with auto-assembling molecules, such as peptides, avoiding issues related to the toxicity of the vector and its functionalization. [165]. These nano-formulations show high selectivity to malignant cells thanks to being activated in specific micro-environments (low pH and the presence of tumor enzymes), reducing the adverse outcomes on healthy tissues [165]. The cut-offs between pro- and antioxidant pathways are the central point in these research fields.

## Figures and Tables

**Table 1:** Biomedical applications of the most frequently used NMs.

Nanomaterial		Biomedical Applications	Ref.
Organic	Liposomes	Drug carrier, fungal and bacterial infections, vaccine formulations, ischemia treatment	[43,44,45,46,47]
	Dendrimers	Antimicrobial and antiviral effects, encapsulation and covalent binding of compounds, tissue engineering, imaging	[48,49,50,51,52,53]
	Carbon-Based	Gene therapy, biosensor, imaging, tissue engineering	[54,55,56,57]
	PLGA/PLA	Imaging, drug delivery	[58,59]
	Protein/Peptide-Based	Target therapy, cancer therapy	[60,61,62]
	Hydrogels	Anti-inflammatory, tissue regeneration, anti-infection	[63,64,65]
	PEG	Drug and Gene delivery	[66]
	Polysaccharide	Drug and Gene delivery, anti-inflammatory, imaging, theranostic medicine	[67,68,69,70]
	Micelles	Cosmetics, drug delivery	[71,72]
	Cyclodextrin-Based	Imaging, anti-cancer drug delivery	[73]
	Solid Lipid/Nanostructured Lipid	Drug loading, diagnostics, RNA delivery, cancer therapies	[74,75]
	DNA Nanoparticles	Imaging, drug delivery	[76]
Inorganic	Gold (Au)	Biosensor, phototherapy, drug delivery	[77,78,79]
	Silver (Ag)	Antiviral, antifungal, antiparasitic, antifouling, wound healing, cancer treatment, drug delivery, dentistry	[80,81,82,83,84,85,86,87]
	Zinc (Zn)	Imaging, drug delivery, diagnostics	[88,89,90]
	Ceria (Ce)	Antioxidant, anti-inflammatory, antiviral	[91,92,93]
	Titanium (Ti)	Antibacterial, cosmetics	[94,95]
	Quantum Dots (QD)	Imaging, drug carrier	[96,97]
	Mesoporous Silica NMs	Drug delivery, imaging, theranostics, tissue engineering, gene therapy	[98]
